# Body mass and cell size shape the tolerance of fishes to low oxygen in a temperature‐dependent manner

**DOI:** 10.1111/gcb.16319

**Published:** 2022-07-25

**Authors:** Wilco C. E. P. Verberk, Jeroen F. Sandker, Iris L. E. van de Pol, Mauricio A. Urbina, Rod W. Wilson, David J. McKenzie, Félix P. Leiva

**Affiliations:** ^1^ Department of Animal Ecology and Physiology Radboud Institute for Biological and Environmental Sciences Radboud University Nijmegen Nijmegen The Netherlands; ^2^ Departamento de Zoología, Facultad de Ciencias Naturales y Oceanográficas Universidad de Concepción Concepción Chile; ^3^ Instituto Milenio de Oceanografía (IMO) Universidad de Concepción Concepción Chile; ^4^ Biosciences, University of Exeter Exeter UK; ^5^ MARBEC, University of Montpellier, CNRS, IFREMER, IRD Montpellier France

**Keywords:** climate change, freshwater, genome size, hypoxia, marine, metabolic scaling

## Abstract

Aerobic metabolism generates 15–20 times more energy (ATP) than anaerobic metabolism, which is crucial in maintaining energy budgets in animals, fueling metabolism, activity, growth and reproduction. For ectothermic water‐breathers such as fishes, low dissolved oxygen may limit oxygen uptake and hence aerobic metabolism. Here, we assess, within a phylogenetic context, how abiotic and biotic drivers explain the variation in hypoxia tolerance observed in fishes. To do so, we assembled a database of hypoxia tolerance, measured as critical oxygen tensions (*P*_crit_) for 195 fish species. Overall, we found that hypoxia tolerance has a clear phylogenetic signal and is further modulated by temperature, body mass, cell size, salinity and metabolic rate. Marine fishes were more susceptible to hypoxia than freshwater fishes. This pattern is consistent with greater fluctuations in oxygen and temperature in freshwater habitats. Fishes with higher oxygen requirements (e.g. a high metabolic rate relative to body mass) also were more susceptible to hypoxia. We also found evidence that hypoxia and warming can act synergistically, as hypoxia tolerance was generally lower in warmer waters. However, we found significant interactions between temperature and the body and cell size of a fish. Constraints in oxygen uptake related to cellular surface area to volume ratios and effects of viscosity on the thickness of the boundary layers enveloping the gills could explain these thermal dependencies. The lower hypoxia tolerance in warmer waters was particularly pronounced for fishes with larger bodies and larger cell sizes. Previous studies have found a wide diversity in the direction and strength of relationships between *P*_crit_ and body mass. By including interactions with temperature, our study may help resolve these divergent findings, explaining the size dependency of hypoxia tolerance in fish.

## Graphical abstract

Whether fish can tolerate low levels of dissolved oxygen is shown here to depend on characteristics of both the fish (body mass, genome size and metabolism) and the water (temperature and salinity). These effects did not act in isolation: In warmer waters, small fishes with small genomes were more tolerant than large fishes with large genomes. We also observed a greater tolerance in freshwater fishes, compared to marine fishes. These findings can help to (i) resolve the scientific debate about oxygen limitation and (ii) predict the impacts of climate change on global fish populations and fisheries.

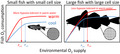

## 1 INTRODUCTION

Like animals in general, fishes rely on sufficient oxygen in their environment to fuel their aerobic energy metabolism by mitochondrial oxidative phosphorylation, which generates 15–20 times more energy (ATP) than anaerobic metabolism (Hochachka & Somero, 2002). However, meeting requirements for aerobic metabolism can be challenging for water‐breathing ectotherms, including fishes (Rubalcaba et al., 2020; Verberk et al., 2011). This is because water holds less oxygen and the rate of oxygen diffusion in water is much lower than that in air (Dejours, 1981). Hypoxia occurs when low environmental oxygen limits the rate of oxygen uptake by a fish, leaving it unable to meet its metabolic oxygen demand (Claireaux & Chabot, 2016). In many aquatic habitats worldwide, hypoxic episodes are increasing in frequency, extent and duration due to anthropogenic global change (Breitburg et al., 2018; Jane et al., 2021; Stramma et al., 2008), posing a threat to fish worldwide.

Hypoxia tolerance, or the ability of an animal to cope with low environmental oxygen, can be measured as the critical oxygen tension (*P*_crit_, sometimes also referred to as limiting oxygen level, LOL), which is defined as the lowest oxygen level at which aerobic metabolism is independent of the ambient partial pressure of oxygen (Grieshaber et al., 1994; Harrison et al., 2018; Hochachka & Somero, 2002; Urbina & Glover, 2013). Under declining oxygen levels and down to *P*_crit_, fishes can sustain oxygen uptake rates to fuel their standard metabolic rate (SMR), the minimum rate of oxygen uptake needed to sustain life in a resting, post‐absorptive state at a given temperature. At *P*_crit_, the maximum capacity to extract oxygen is presumably engaged, in terms of gill ventilation and perfusion, just to sustain this minimum uptake rate (Kielland et al., 2019; Seibel & Deutsch, 2020). Below *P*_crit_, rates of oxygen uptake are lower than SMR and fishes must either generate energy anaerobically or suppress their metabolism.

*A* species' hypoxia tolerance can be used to infer its aerobic scope and hence its energy budgets (Seibel & Deutsch, 2020) and relates to habitat utilization (Deutsch et al., 2020). Uncovering general patterns in fish hypoxia tolerance is therefore valuable not only from a theoretical point of view; this also has an applied value. Here, we contribute to this objective by investigating which abiotic and biotic factors explain the observed variation in hypoxia tolerance across fish species. As hypoxia tolerance relates to a mismatch between oxygen supply and demand, we focus on factors that relate to supply and demand. Any environmental factor that influences either oxygen demand or availability can impact hypoxia tolerance and, therefore *P*_crit_. For example, water temperature affects both the availability of oxygen and the metabolic demand for oxygen (Verberk et al., 2011), and *P*_crit_ has been reported to vary with temperature (Deutsch et al., 2020; Rogers et al., 2016). Environmental salinity is also relevant because gills are a major site for ion exchange (Randall et al., 1972). The energy costs of moving ions against their prevailing gradient range from virtually zero to one‐third of standard metabolism (Ern et al., 2014). Furthermore, fish hyperventilate in hypoxia to maintain oxygen uptake rates, which reduces gill boundary layers and enhances passive ion movements (losses in freshwater and gains in seawater). This could magnify energetic costs for active ion transport in the opposite direction. At the same time, oxygen availability decreases with increasing salinity due to the decline in oxygen solubility (Verberk et al., 2011). Finally, there could be a stronger selection for increased hypoxia tolerance in freshwater compared to marine fish, as freshwater habitats are typically more prone to hypoxic episodes.

Since the pioneering work of Prosser (Prosser, 1955), there has been debate in the literature about the size‐dependency of hypoxia tolerance in fish. Body mass profoundly influences oxygen consumption rates in fishes, both within species (during ontogeny) and across species (Clarke & Johnston, 1999; Killen et al., 2010; Rubalcaba et al., 2020; Urbina & Glover, 2013). Both the capacity for oxygen supply to tissues and metabolic oxygen demand scale with body mass, but if the exact scaling relationship is different, a mismatch in supply and demand occurs such that hypoxia tolerance should vary with size (Deutsch et al., 2020; Killen et al., 2016; Rubalcaba et al., 2020; Urbina & Glover, 2013). However, several studies comparing species report no clear effect of mass on *P*_crit_ in fishes (Nilsson & Östlund‐Nilsson, 2008; Seibel & Deutsch, 2020). Rogers et al. (2016) investigated the effects of body mass and several environmental parameters and found a positive relationship between fish size and *P*_crit_ (i.e. hypoxia tolerance decreased with fish size). A problem, however, with disentangling an effect of body mass in many physiological patterns is that variation in body mass across species is phylogenetically structured and covaries with many other morphological and life‐history aspects of a species. Thus, species that share an evolutionary history may be similar in both body mass and *P*_crit_, potentially resulting in spurious correlations if phylogeny is not considered. In our investigation, we used Bayesian phylogenetic models to account for evolutionary history and test whether *P*_crit_ is phylogenetically structured (i.e. whether hypoxia tolerance is more similar among closely related species). We also included traits co‐varying with body mass to disentangle the effects of body mass from other possible causal factors, such as the metabolic rate and cell size.

On average, larger‐bodied fishes tend to be composed of larger cells, and cell size affects oxygen supply and demand (Kozłowski et al., 2020; Maciak et al., 2011). Larger cells result in lower metabolic costs as the lower membrane surface area to volume ratio reduces the costs for phospholipid turnover and maintenance of transmembrane ion gradients. In contrast, smaller cells can better provision oxygen to mitochondria as intracellular diffusion distances are shorter and oxygen diffuses more rapidly in lipid membranes than in aqueous cytosol (Subczynski et al., 1989; Szarski, 1983). Although cell size is not exhaustively documented for fishes, there is a tight correlation between the cell size of a fish species and the size of its genome (Gregory, 2001; Malerba & Marshall, 2021), making it possible to use genome size as a *proxy* for cell size. Gregory (2001) compared fish species with genome sizes spanning two orders of magnitude and reported a more than 10‐fold increase in cell size associated with this difference in genome size.

There is evidence that the effect of both body mass and cell size on rates of oxygen consumption is dependent on water temperature (Hermaniuk et al., 2021; Killen et al., 2010; Rubalcaba et al., 2020). The increased metabolic rates typically observed in warmer water are reported to be more pronounced in smaller fishes (Rubalcaba et al., 2020) and fishes composed of larger cells are able to maintain higher metabolic rates in the cold (Hermaniuk et al., 2021). For these reasons, we hypothesize that hypoxia tolerance is related to body mass and cell size in a temperature‐dependent manner. We test whether differences in hypoxia tolerance can be better explained by models including the interaction with temperature and, if so, whether the interaction is such that the effect of warming on hypoxia tolerance is more pronounced in fishes with a larger body mass and genome size.

## 2 METHODS

### 2.1 Compilation of *P*_crit_ data

A pre‐existing database on fish *P*_crit_ (Rogers et al., 2021) was expanded with data from papers published between January 2016 and March 2021. New papers were sourced from the Web of Science Core Collection using the following keyword combinations of Boolean terms: (*P*‐crit or critical oxygen tension or loss of equilibrium or limiting oxygen) and (fish or teleost or bony fish). After an initial screening of the title and abstract to identify papers that potentially had data on *P*_crit_ as a proxy for hypoxia tolerance, 81 papers remained. These papers were read in detail to extract and add data from 55 additional papers to the database, yielding 350 additional records for 72 species. A list of the data sources is provided in Appendix S1. To allow comparison and analysis of the data, units of metabolic rate and *P*_crit_ were recalculated to μmol O_2_ h^−1^ and kPa, respectively. *P*_crit_ values were converted to kPa using the web‐based tool “loligo oxygen converter” (May 2021), which accounts for the effect of temperature and salinity on oxygen solubility (Verberk et al., 2011), and in which atmospheric pressure was set to 760 mmHg representing a standard atmospheric pressure at sea level. If salinity was only specified qualitatively as ‘sea water’ in the paper, salinity was set to the average ocean salinity of 35 PSU.

In addition to testing whether *P*_crit_ related to the body mass of the individual fish, we also wanted to test whether *P*_crit_ varied across ontogeny. To this end, we expressed the individual body mass as a percentage of that species' known maximum body mass. Thus, a fully grown individual of a small species would yield a high percentage, while a small juvenile of a large species would yield a low percentage, even though these individuals could be similar in size in absolute terms. We gathered published body length data from FishBase (June 2021) and converted this to maximum body mass using the length‐weight relationship available on FishBase (June 2021). If the length‐weight relationship of a species was not available, the relationship of a closely related species (mostly in the same genus) was used. To test the validity of using conversion indices from closely related species, we used another closely related species to get a second estimate for maximum body mass and found that both estimates were strongly correlated (*R* ^2^ = .987; Figure S1).

As a proxy for cell size, we used genome size data (*C*‐value; the mass of DNA in a haploid set of chromosomes in picograms, 1 pg ≈ 978 million base pairs of DNA), collected from the online database (Gregory, 2021, accessed on June 2021). Genome size data obtained using the flow cytometry method (FCM, mostly on erythrocytes) were preferentially collected. In the case of multiple entries for a given species, genome sizes were averaged.

### 2.2 Phylogeny

In ectotherms, it is well known that a shared evolutionary history among species can influence variation in physiological traits (Leiva et al., 2019; Rezende & Diniz‐Filho, 2012). Closely related species tend to be more similar in traits and should not be considered independent elements of evolution in comparative analyses. For such reasons, we included the phylogenetic relationships of the fish species in our statistical models as a variance–covariance matrix. Before retrieving information on the phylogenetic relationships, we corrected the scientific names of the last version of our database, dated June 2021. The database contained 214 entries of taxa at the taxonomic rank up to the species and subspecies level. A visual inspection for syntax mistakes (e.g. *Ctenopharyngodon idella* instead of *Ctenopharyngodon idellus*) or wrongly labelled species was performed. Scientific names were searched in the Open Tree of Life (OTL, https://tree.opentreeoflife.org) and retained up to the level of species, with the exception of certain species such as *Cyprinus carpio*, which was aggregated at the subspecies level as *Cyprinus carpio carpio*. Other entries using hybrids (e.g. *Lepomis* hybrid between bluegill and pumpkinseed), with no phylogenetic information (*Rostaraja eglanteria*) or with the flag “incertae_sedis_inherited” (*Chrysiptera flavipinnis* and *Acanthoclinus fuscus*) were excluded. The final phylogenetic tree from the OTL was pruned to include only species present in our database. The final number of taxa names in the database was reduced to 195 unique species (tips labels). Branch lengths were set equal to the number of descendant tips minus one, following the method of Grafen (Grafen, 1989). In addition, preliminary analyses considered transformation effects of Grafen's rho (*ρ*) and found that a phylogenetic tree rescaled by Grafen's rho (*ρ* = 0.4) provided the best fit to the data. Using this transformation effect, branches in a phylogenetic tree near the tips are expanded, so recent evolutionary changes have added weight.

### 2.3 Data analysis

We used a Bayesian phylogenetic multilevel modelling framework (Bürkner, 2017, 2021) to describe *P*_crit_ as a response to body mass, genome size, measurement temperature, salinity, metabolic rates, acclimation temperature and respiratory method (closed respirometry, intermittent flow respirometry and unknown). For 600 records from 171 species, we had data on all of these variables. The metabolic rate was corrected for known effects of temperature and body mass in fishes (Rubalcaba et al., 2020) by taking the residuals of a model with the (log_10_‐transformed) whole‐organism metabolic rate as the response variable and salinity, temperature and (log_10_‐transformed) body mass as the independent variables. The residuals of this model, which explained 93.8% of the variation in the metabolic rate, reflect whether the metabolic rate observed in an individual was higher or lower than expected based on its body mass and water conditions. For species lacking information on the metabolic rate, we set their residual metabolism to 0. We assessed the correlation between our independent variables (Figure S2) and observed strong correlations between variables related to temperature (latitude, measurement temperature, acclimation temperature) and between variables related to body mass (individual body mass, percentage body mass and maximum body mass). For most records, the acclimation temperature is identical to the measurement temperature, but this is not always the case. The expectation would be that warm‐acclimated fish would be better at dealing with hypoxia than cold‐acclimated fish, but only when measured at the same temperature (Collins et al., 2021). To account for the collinearity between acclimation temperature and measurement temperature, we employed a measure of relative acclimation temperature, calculated as the difference between acclimation temperature and measurement temperature. Thus, negative values indicate that fish were acclimated at temperatures cooler than those at which they were tested and vice versa for positive values. Since relative acclimation temperature and measurement temperature were only weakly (negatively) correlated, we could include both in our analyses. For both body mass and genome size, we also included interactions with temperature. To test whether *P*_crit_ varied across ontogeny, we also fitted models that included body mass expressed as a percentage of the maximum mass for that species. Both body mass and percentage of body mass were log_10_‐transformed, and a quadratic term of the latter was included to test for non‐linear relationships. To test if *P*_crit_ responds differently to distinct predictors, we used an information‐theoretic framework and built a set of competing models that tested distinct combinations of variables, including interactions with temperature. In our models, we considered two random effects (varying intercepts). In addition to the variance–covariance matrix of the phylogenetic tree, we included an unstructured random species effect, which accounted for any specific effect that would be independent of the phylogenetic relationship among species. For fixed effects, we used weakly informative priors to achieve good convergence following a Gaussian distribution (two chains of 4000 iterations with 1000‐warm‐up periods). We applied a model selection procedure by adding sequentially the interaction between temperature and the different body mass‐related predictors (body mass, genome size, rescaled body mass and the quadratic term of body mass) and at the same time considering the other variables of salinity, residuals of metabolic rates, relative acclimation temperature and respiratory method. We used the Pareto‐smoothed importance sampling leave‐one‐out (PSIS‐LOO) cross‐validation (Vehtari et al., 2017) to calculate the model weights. This approach allows competing models against one another, assigning the probability of a particular model candidate to a model set. In the end, we dropped any predictor which was not consistently significant in our models as evaluated by a Bayesian equivalent of the *p*‐value based on a Maximum A Posteriori (MAP) Bayesian Hypothesis Testing framework (Makowski et al., 2019). With this approach, we excluded relative acclimation temperature from the final model. In addition, we excluded the type of respirometry from the final model as we did not find a difference between closed and intermittent flow respirometry. Posterior model parameters for the most parsimonious model were estimated using Markov chain Monte Carlo (MCMC) methods by constructing three chains of 15,000 iterations, 7500‐warm‐up periods and total post‐warmup draws of 22,500. Data files and code supporting analyses, figures and tables of this study are publicly available on GitHub (https://github.com/felixpleiva/Hypoxia_tolerance_fishes; Verberk et al., 2022).

To determine the proportion of the variance explained for the different predictors, the total variance in the data was partitioned into marginal (sum of fixed effects without random effects), random effects only (phylogeny and species) and the residual variance using the approach of Gelman et al. (2019). As an equivalent to Pagel's lambda, we calculated the phylogenetic heritability as the proportion of variance, conditioned on the fixed effects, attributable to the random effect of phylogeny (Hadfield & Nakagawa, 2010).

We also visualized the effects of temperature on *P*_crit_ to demonstrate how water temperature differentially affected fishes differing in body mass and genome size. To this end, we used a global map of water temperature obtained by combining global freshwater surface temperature models with freely available sea surface temperature data (Barbarossa et al., 2021). The annual maximum weekly water temperature averaged over 1976–2005 and across five global climate models constitutes the layer for freshwater temperatures. The annual maximum monthly water temperature averaged over 2003–2020 constitutes the layer for sea surface temperature. Both temperature layers were combined with the model output to calculate the *P*_crit_ for contrasting body mass and genome sizes, separately for freshwater and marine fishes. We used 5 g and 1000 g for small and large body mass, respectively, and 0.5 pg and 4 pg for small and large genomes, respectively. The freshwater and marine layer were subsequently combined. The calculated values for *P*_crit_ were then converted into a measure of factorial aerobic scope (FAS) by assuming that SMR can increase proportionally with environmental oxygen tension, following equation: FAS = 21/*P*_crit_ (Seibel & Deutsch, 2020). Calculated *P*_crit_ values below 2 (for instance at extremely cold temperatures for large fish) were set to 2, constraining maximum FAS to 10.5.

All analyses were carried out in R version 4.1.0 by using the following packages: “brms” (Bürkner, 2017, 2018), “ape” (Paradis & Schliep, 2019), “dplyr v1.0.7” (Wickham et al., 2021), “stringr v1.4.0” (Wickham, 2019), “phytools” (Revell, 2012), “tidybayes v3.0.1” (Kay, 2021), “bayestestR” (Makowski et al., 2019), “geiger” (Harmon et al., 2008; Pennell et al., 2014), “caper v1.0.1” (Orme et al., 2018), “rotl” (Michonneau et al., 2016), “ggplot2” (Wickham, 2016), “ggtree” (Yu et al., 2017), “treeio” (Wang et al., 2020) and “purrr” (Henry & Wickham, 2020).

## 3 RESULTS

The most parsimonious model (Table 1) explained around 80.5% (credible interval CI, 78.6–82.2) of the variation in critical oxygen tension (*P*_crit_), which is used as a proxy for hypoxia tolerance. Variation in *P*_crit_ could be explained by phylogeny (38%), differences across species independent of phylogeny (12%), and the marginal effects included in the model (salinity, residuals of metabolic rate, body mass, genome size and their interactions with temperature, which together explained 31%). Our results indicate that tolerance to hypoxia is conserved phylogenetically in fishes (equivalent lambda = 0.56 [CI 0.30–0.78]). Much of the variation in *P*_crit_ was shared between the marginal effects and the effects of species phylogeny (Figure S3), reflecting phylogenetically structured adaptation, likely arising as closely related species have experienced similar selection pressures.

**TABLE 1:** Average estimates, 95% posterior credible intervals and effective sampling of Bayesian posterior distributions for fitted parameters of the most parsimonious model. Bayesian *p*‐value based on the density at the maximum a posteriori (MAP) are also given.

Parameter	Mean estimate	2.5%	97.5%	Effective sampling	*p*(MAP)
Random effects					
Species	1.02	0.18	1.57	1157	
Phylogeny	2.22	1.40	3.11	1471	
Fixed effects					
Intercept	5.56	3.56	7.59	9599	<.001
Measurement temperature, *T*	−0.03	−0.08	0.02	11,894	.512
Log_10_ genome size (GS)	−7.47	−11.11	−3.87	11,618	<.001
Log_10_ body mass (BM)	−1.37	−1.99	−0.74	11,312	<.001
Measurement salinity	0.08	0.06	0.09	10,384	<.001
Residuals of metabolic rate	2.20	1.27	3.14	17,375	<.001
*T* × GS	0.44	0.30	0.58	10,840	<.001
*T* × BM	0.06	0.03	0.08	11,437	<.001

**FIGURE 1:**
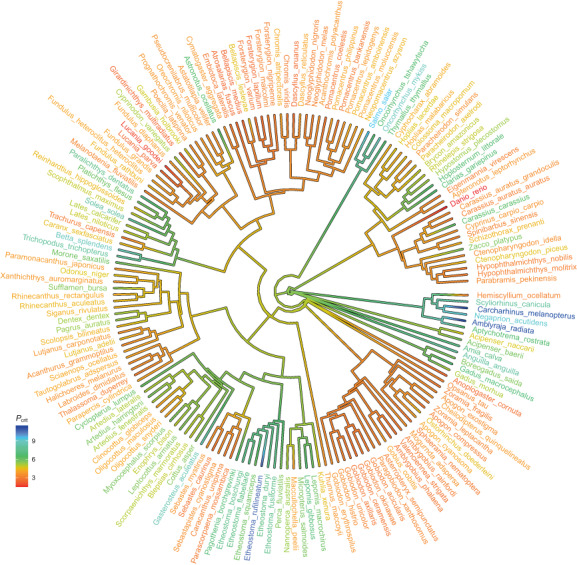
The phylogeny of the 171 fish species analysed in our study. Colours reflect variation in *P*_crit_ from low values in red (hypoxia tolerant) to high values in blue (hypoxia sensitive). Variation in *P*_crit_ exhibited a clear phylogenetic signal.

Cartilaginous fishes were among the most sensitive to hypoxia, although data on *P*_crit_ were only available on six species in this group (Figure 1). Within the bony fishes, the order Cyprinodontiformes and the family Cyprinidae were among the most tolerant to hypoxia. In addition, species was included as a random effect to account for the non‐independence of multiple measures *per* species. This random effect was also significant (i.e. confidence intervals did not overlap 0, see Table 1). This effect likely reflects consistent tolerance characteristics that are intrinsic to the ecology of an individual species, but which are not captured by phylogeny or any of the other species‐specific variables (see below).

**FIGURE 2:**
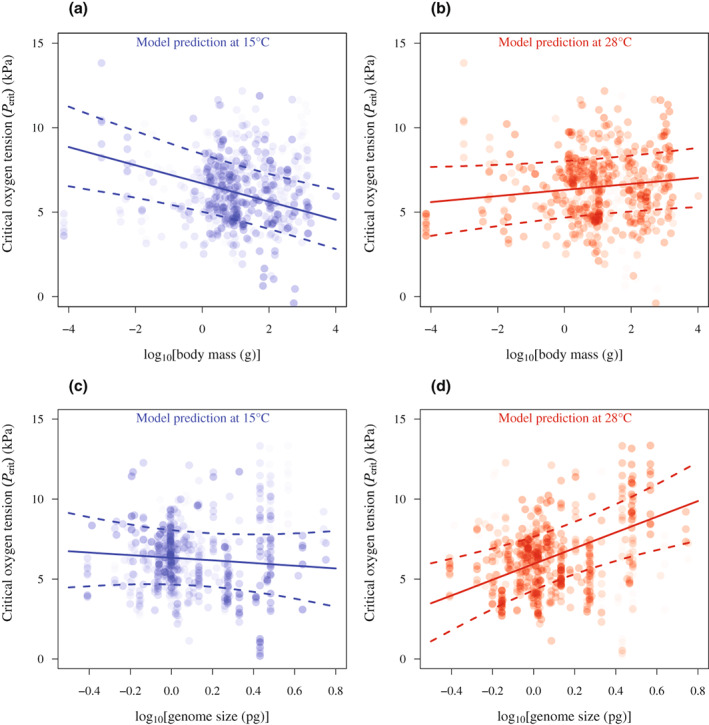
Effects of body mass and genome size on critical oxygen tension (*P*_crit_). The plots show the partial residuals, with predictions from the model for 15°C (a, c; blue) and 28°C (b, d red). These temperatures reflect the 25th and 75th percentile, respectively. The data points are identical for the plots of body mass (a, b) and plots of genome size (c, d). However, individual data points differ in their transparency, reflecting the water temperature at which *P*_crit_ was measured (in a, c, less transparent data points approximate 15°C; in b, d, less transparent data points approximate 28°C).

We found interactive effects between temperature and both genome size and body mass (Figure 2). Fishes with a larger body mass or genome size were more susceptible to hypoxia in warmer water but less susceptible in colder water, compared to fishes with a smaller body mass or genome size. Genome size was tightly linked to cell size, but fishes with larger genomes also tended to be larger. In our dataset, genome size was weakly correlated to the size of the fish (Figures S2 and S4), but no correlation was found between body mass and genome size when analyzed within a phylogenetic context (*p* > .668). In addition, a model explaining variance in *P*_crit_ across fishes, which included maximum body mass instead of genome size, received far less support (Table S2). Thus, the effects of genome size are likely to reflect the effects of cell size rather than being confounded by maximum body mass.

**FIGURE 3:**
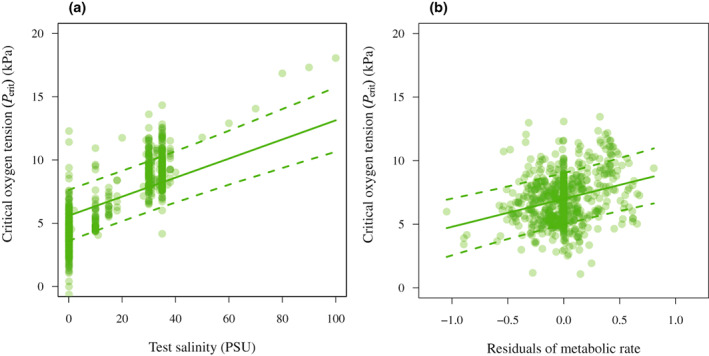
Variation in *P*_crit_ as a function of salinity (a) and residual metabolic rate (b). Plotted are partial residuals, with predictions from the model (Table 1).

Freshwater fishes were more tolerant to hypoxia than marine fishes, with *P*_crit_ values being about 2‐fold lower at the highest salinities (Figure 3a). We also checked if the model estimate for salinity changed when excluding records for which we only had qualitative data on salinity (labelled as ‘seawater’). However, the estimate for the full model was similar to that of the reduced model excluding these records (full model: 0.0755, range 0.0562–0.0949; reduced model: 0.0782, range 0.0590–0.0976). Furthermore, fishes with relatively high oxygen requirements (residual metabolic rate, accounting for the known effects of temperature and body mass) were more sensitive to hypoxia (Figure 3b).

**FIGURE 4:**
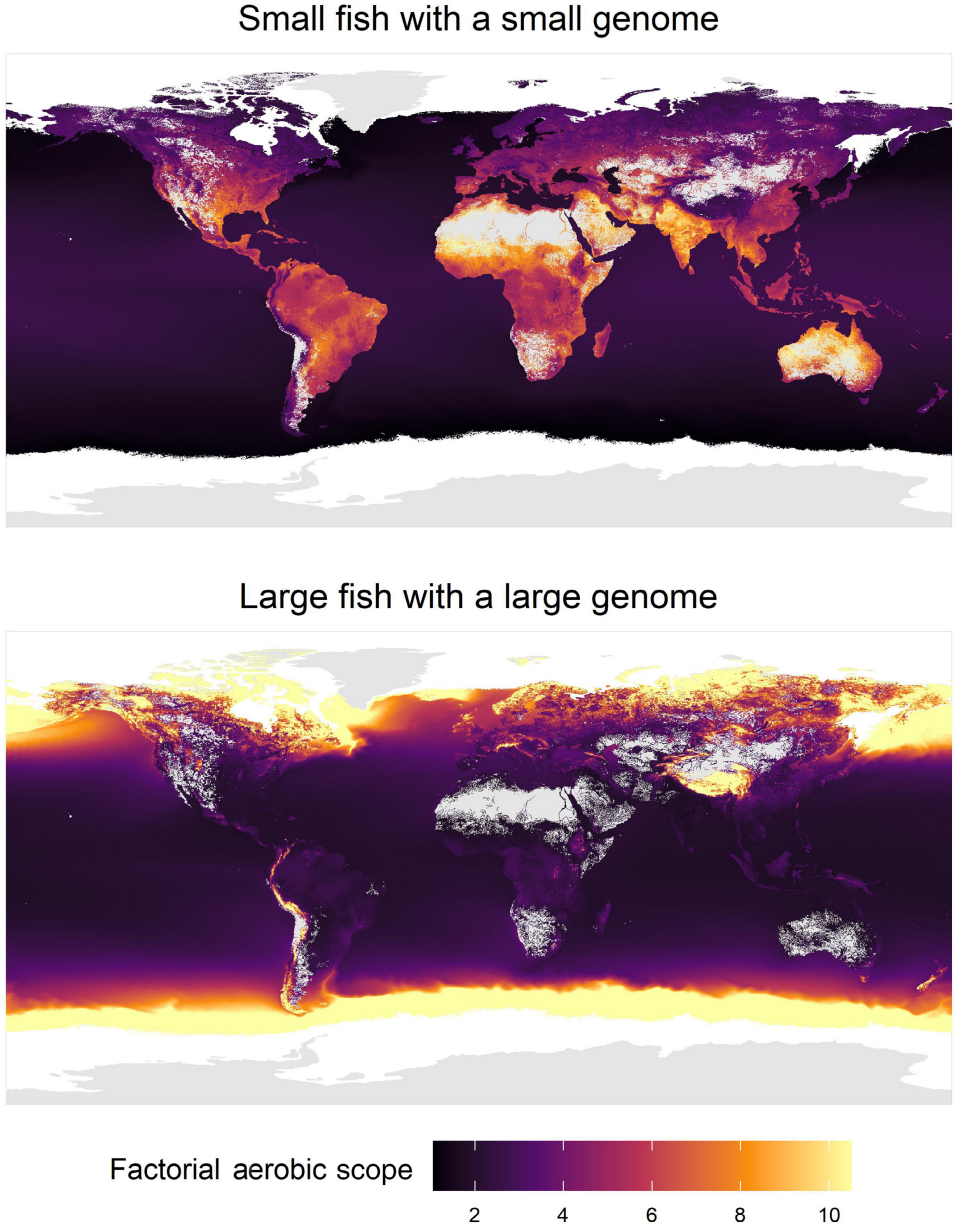
Global variation in factorial aerobic scope as a function of water temperature. Global temperature maps were converted for two cases. Upper panel: Fishes with a low body mass (5 g) and a small genome (*C*‐value: 0.5 pg). Lower panel: Fishes with a high body mass (1000 g) and a large genome (*C*‐value: 4 pg).

The interactive effects between temperature and the body mass and cell size of fishes have repercussions for the conditions under which fishes can maintain a high aerobic scope necessary to be active, grow and reproduce (Figure 4). For example, large fishes with large genomes will exhibit high factorial aerobic scope in cold, polar water and they will quickly lose aerobic scope in warmer waters. In contrast, small fishes with small genomes will exhibit the highest factorial aerobic scope in warm, equatorial waters.

## 4 DISCUSSION

**FIGURE 5:**
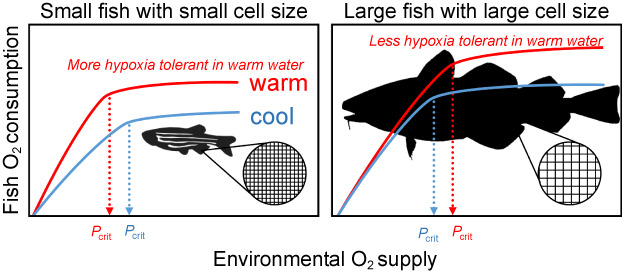
Conceptual diagram illustrating the interactions between body mass, cell size, temperature, oxygen, and hypoxia tolerance (*P*_crit_). Temperature has a different effect on the *P*_crit_ of fish species with a small body mass and small cells (owing to their small genome) compared to fishes with a large body mass and large cells (owing to their large genome). Temperature increases both oxygen supply and consumption, but in small fishes, the effects on supply are stronger, leading to a better hypoxia tolerance (lower *P*_crit_). In contrast, in large fishes, the effects on consumption are stronger, leading to a greater hypoxia sensitivity (higher *P*_crit_). Credits for (a) *Danio rerio* silhouette by Jake Warner. Credits for (b) *Gadus morhua* silhouette by Milton Tan. Both silhouettes are available for reuse on PhyloPic (www.phylopic.org) under the Public Domain Dedication 1.0 license.

Elevated temperatures and hypoxia are two important stressors for fishes and aquatic life in general, and they are projected to become more prevalent and intense in the near future. Furthermore, they are likely to co‐occur because in warmer water, less oxygen is dissolved, while increased metabolism results in enhanced rates of oxygen depletion (Rubalcaba et al., 2020). Previous studies report lower hypoxia tolerance in fishes, reflected by increased *P*_crit_ values, at increasing temperatures (Deutsch et al., 2020; Rogers et al., 2016). This indicates that when warming and hypoxia co‐occur, they act synergistically in limiting species distributions (Deutsch et al., 2020; Verberk et al., 2016). Here, we expand on that by showing that the reduction in hypoxia tolerance in warmer water was particularly pronounced for large‐bodied fishes and for fishes with large genomes (Figure 5).

Several factors govern the rate at which oxygen can be extracted from the water, the most important ones being gill surface area, the rate at which the gills are ventilated and patterns of gill perfusion (e.g. Bigman et al., 2021; Randall, 1982; Rubalcaba et al., 2020). Our results point to additional factors controlling the rate of oxygen extraction in a size and temperature‐dependent manner, such as the boundary layer at the gill epithelium. Higher oxygen requirements in warmer waters will cause oxygen depletion in the boundary layer at the gill epithelium more readily, providing a feedback mechanism that makes oxygen uptake increasingly difficult. Drawing on data on fish oxygen consumption rates, Rubalcaba et al. (2020) suggested that this feedback mechanism may result in oxygen becoming limiting even in normoxic water, especially when fishes are large, active and in warm water (Rubalcaba et al., 2020). In hypoxic waters, oxygen could even become limiting for inactive fishes. Here, we show that when analysing hypoxia tolerance (*P*_crit_) directly, a similar pattern emerges whereby hypoxia tolerance of larger fishes is reduced in warmer water (higher *P*_crit_). In contrast, very small fishes (<1 g) were found to increase their hypoxia tolerance in warmer waters (lower *P*_crit_; compare Figure 2a,b). The thickness of the boundary layer at the gill epithelium varies with the temperature of water via effects on viscosity and with body mass via effects on Reynolds numbers (Rubalcaba et al., 2020; Verberk & Atkinson, 2013). Colder water is more viscous and this may impede gill water convection proportionally more in small fishes, such that the water in the boundary layer may become more easily depleted of oxygen, especially when oxygen in the bulk water is limited (i.e. under hypoxia). Conversely, the decreased viscosity of warmer water facilitates water convection, which enables small fishes to maintain oxygen uptake rates in hypoxia. The best model did not include percentage body mass (expressing the actual body mass as a percentage of the maximum body mass for a given species, see Section 2), suggesting that the absolute body mass is what matters here.

We used genome size as *a proxy* of cell size to evaluate the effects of cell size on *P*_crit_, since genome size is tightly correlated with cell size in fishes (e.g. Gregory, 2001; Maciak et al., 2011; Malerba & Marshall, 2021; Pol et al., 2020). However, genome size also covaries with body mass. In our dataset, the correlation between both was weak (Spearman rank: rho = 0.197, *p* < .001), making it possible to include both body mass and cell size in a single model. Also, a model that substituted genome size for maximum body mass received less support (Table S2), indicating that the effects of genome size are most likely due to cell size. Larger cells have a lower membrane surface area to volume ratio and longer intracellular diffusion pathways (Atkinson et al., 2006; Kozłowski et al., 2020; Miettinen et al., 2017; Szarski, 1983; Woods, 1999). All other things being equal (e.g. cell shape and positioning of the mitochondria), larger cells are therefore expected to have a lower capacity for oxygen uptake and oxygen delivery to the mitochondria. A specific example of this diffusion distance issue that has importance for whole animal gas exchange is that, in hypoxia, oxygen may bind more rapidly to haemoglobin in small red blood cells during passage through the gills. At high temperatures, when demand for oxygen is stimulated, fish species with a smaller cell size (owing to their smaller genome) did indeed have greater hypoxia tolerance (lower *P*_crit_ values; Figure 2d). Conversely, fishes with larger cells (owing to their larger genome) exhibited improved hypoxia tolerance at colder temperatures. This matches previous work on triploid fish with larger cells, which were found to be capable of maintaining higher rates of oxygen consumption than their diploid counterparts, but only at the colder temperature (Atkins & Benfey, 2008; Hermaniuk et al., 2021). Our results indicate that a better performance in the cold of fishes with large cells also extends to hypoxia tolerance (lower *P*_crit_).

Efficient oxygen delivery from the water to the mitochondria requires capacities to be matched across the oxygen cascade from the uptake capacity of the ventilatory system to the transport capacity of the circulatory system, down to the capacity of mitochondria to metabolize oxygen and generate ATP. Our finding that both body mass and genome size shape hypoxia tolerance in interaction with temperature suggests that this cascade has rate‐limiting steps at both the whole‐organism level and the cellular level. At colder temperatures, the difference in *P*_crit_ is the greatest between large and small‐bodied fishes and so we hypothesize that rate‐limiting steps in the oxygen cascade would be related to the efficiency at which fishes can ventilate their gills to promote diffusion from water to blood, which is size‐dependent, especially in cold, viscous water (Verberk & Atkinson, 2013). At warmer temperatures, the difference in *P*_crit_ is largest between fishes differing in cell size and so we hypothesize that rate‐limiting steps could be related to oxygen transport by erythrocytes or constraints on oxygen diffusion into respiring cells. This suggests that even though the capacity of the different steps in the oxygen cascade may be generally matched through natural selection as embodied in the principle of symmorphosis (Weibel et al., 1991), oxygen delivery from the environment to mitochondria may still encounter rate‐limiting steps at different levels, ranging from the gills to the cellular level, depending on the temperature.

Compared to freshwater, the availability of oxygen is reduced in saline water (Dejours, 1981; Verberk et al., 2011), and this could explain at least partly the increased hypoxia tolerance exhibited by freshwater fishes as demonstrated here and elsewhere (Figure 3a; Rogers et al., 2016; Seibel & Deutsch, 2020). After accounting for the reduced availability of oxygen in saline water, the effect of salinity decreases (model estimates drop from 0.08 to 0.03; Table 1), but remains highly significant (*p* < .001). Thus, the ecology and evolution of these fishes may also play a role. Freshwater animals probably experience greater fluctuations in oxygen levels, especially in small, stagnant and warm waters near the equator. In addition, owing to the small volume of freshwater systems, greater fluctuations in temperature can be expected, including episodes of high temperatures. Acute warming imposes high metabolic demands on freshwater fishes, and these may have selected for an enhanced capacity to supply sufficient oxygen (e.g. Harter et al., 2022 report greater haemoglobin‐oxygen binding affinities in freshwater fishes), resulting in lower *P*_crit_ values. We indeed observed that fishes with relatively high oxygen requirements (residual metabolic rate, accounting for the known effects of temperature and body mass) were more sensitive to hypoxia (Figure 3b). Similar findings have been reported for sculpins (Mandic et al., 2008). Since most *P*_crit_ experiments are based on SMR, a higher (residual) metabolism here means higher oxygen requirements, rather than increased oxygen supply capacity, explaining the observed relationship.

Oxygen limitation and its dependence on body mass and temperature have been debated in the literature, with discussions focusing on the mass scaling exponents of oxygen consumption rates (Killen et al., 2016; Rubalcaba et al., 2020), whether or not oxygen uptake is limiting at the gills (Lefevre et al., 2017; Pauly, 2021), and whether oxygen limitation can explain effects of temperature on body mass (Audzijonyte et al., 2020; Verberk et al., 2021 ). A size‐dependency of hypoxia susceptibility has also been invoked to explain the size‐dependency of thermal tolerance in aquatic ectotherms (Dahlke et al., 2020; Leiva et al., 2019). Here, we have demonstrated that whether larger fishes are more sensitive to hypoxia is conditional upon the prevailing temperature. These interactive effects with temperature can explain why previous studies have not found a clear relationship between *P*_crit_ and body mass. Therefore, our study helps resolve the debate about the size dependency of hypoxia tolerance in fishes.

Our findings are consistent with the general observations of the temperature‐size rule, whereby aquatic ectotherms such as fishes grow to smaller body sizes when reared under warmer temperatures (Loisel et al., 2019). One of the mechanisms proposed to explain the temperature‐size rule involves a potential oxygen limitation becoming more pronounced in warmer water and in animals that are larger or have larger cells (Verberk et al., 2021). Although the temperature‐size rule is an intraspecific pattern, the magnitude of size responses to temperature varies across species. Aquatic ectotherms exhibit stronger body size reductions in response to warming than terrestrial ectotherms (Horne et al., 2015) and this has been taken as evidence in support of the oxygen limitation mechanism as the availability of oxygen in water is lower than that in air. Our results of greater hypoxia sensitivity in larger fishes and in fishes with larger cells in warm water, match the stronger size reductions to warming observed in larger species and in species with larger cells (Verberk et al., 2021). Our findings are also consistent with the observation that species with larger genomes are preferentially distributed in colder waters (Dufresne & Jeffery, 2011). In addition to respiration (a loss of energy), growth and body mass are also dependent on feeding (a gain in energy; e.g. Lindmark et al., 2022). Given that hypoxia has been shown to reduce appetite in fish (Bernier et al., 2012), and that fish may reduce their meal size in warm waters to retain sufficient aerobic scope for digestion and other aerobic functions (Jutfelt et al., 2021), the size and temperature dependency of hypoxia tolerance demonstrated here may also impact growth performance by modulating fish appetite and feeding.

In conclusion, especially at high temperatures, larger fishes and fishes with larger cells appear to be more sensitive to hypoxia. Although the different steps of the oxygen cascade have likely co‐evolved, it is possible that the rate‐limiting step shifts with temperature. In cold viscous water, efficient gill perfusion may be energetically costly, constraining oxygen uptake, especially in smaller fishes. In warmer waters, meeting the increased energy and hence oxygen requirements at the cellular level may be constrained by oxygen diffusion across cell membranes, especially in fishes with larger cells.

A consequence of the thermal dependencies demonstrated here is that across global clines in water temperature, aerobic scope and hence energy budgets will differ substantially for fishes of different body mass and cell size and between freshwater and marine species (Figure 4). This adds a new size dimension to the previous demonstrations that temperature and oxygen interactively constrain the geographic distribution of aquatic ectotherms (Deutsch et al., 2020). Our study details how the aerobic scope of fishes differing in body mass and cell size is likely to be impacted by warming. This can be used to predict how fishes will shift their geographic distribution ranges in a size‐dependent fashion. Moreover, even if the geographic distribution is unaltered, growth performance and the size structure of fish populations may be affected by warming. Since body mass has disproportionate effects on fish fecundity (Barneche et al., 2018), this knowledge has important implications for refining the precision of models used to predict the impacts of climate change on global fish populations and fisheries (Cheung et al., 2010; Marshall et al., 2021).

## CONFLICT OF INTEREST

The authors declare no competing interests.

## Supporting information


Appendix S1

Appendix S2

## Data Availability

Data files and code supporting analyses, figures and tables of this study are publicly available in GitHub Repository (https://github.com/felixpleiva/Hypoxia_tolerance_fishes). When using the data or code from this project, please cite https://doi.org/10.5281/zenodo.6123770.
