# Supplementary material for: Body mass and cell size shape the tolerance of fishes to low oxygen in a temperature‐dependent manner
Source: Glob Chang Biol. 2022 Jul 25;28(19):5695–707. doi: 10.1111/gcb.16319 (PMC9542040; doi:10.1111/gcb.16319)
Supplement: Supplementary file 1 — Appendix S1 [file GCB-28-5695-s001.docx]

Appendix 1. list of the data sources.

Babikian, J., Nasser, N., Monzer, S., & Saoud, I. P. (2017). Survival and respiration of marbled rabbitfish (Siganus rivulatus) fingerlings at various oxygen tensions. Aquaculture Research, 48(8), 4219–4227.

Barnes, R. K., King, H., & Carter, C. G. (2011). Hypoxia tolerance and oxygen regulation in Atlantic salmon, Salmo salar from a Tasmanian population. Aquaculture, 318(3–4), 397–401. https://doi.org/10.1016/j.aquaculture.2011.06.003

Belão, T. C., Leite, C. A. C., Florindo, L. H., Kalinin, A. L., & Rantin, F. T. (2011). Cardiorespiratory responses to hypoxia in the African catfish, Clarias gariepinus (Burchell 1822), an air-breathing fish. Journal of Comparative Physiology B, 181(7), 905–916.

Berschick, P., Bridges, C. R., & Grieshaber, M. K. (1987). The influence of hyperoxia, hypoxia and temperature on the respiratory physiology of the intertidal rockpool fish Gobius cobitis Pallas. Journal of Experimental Biology, 130(1), 368–387.

Borowiec, B. G., Crans, K. D., Khajali, F., Pranckevicius, N. A., Young, A., & Scott, G. R. (2016). Interspecific and environment-induced variation in hypoxia tolerance in sunfish. Comparative Biochemistry and Physiology Part A: Molecular & Integrative Physiology, 198, 59–71.

Borowiec, B. G., Hoffman, R. D., Hess, C. D., Galvez, F., & Scott, G. R. (2020). Interspecific variation in hypoxia tolerance and hypoxia acclimation responses in killifish from the family Fundulidae. Journal of Experimental Biology, 223(4), jeb209692.

Bouyoucos, I. A., Trujillo, J. E., Weideli, O. C., Nakamura, N., Mourier, J., Planes, S., Simpfendorfer, C. A., & Rummer, J. L. (2021). Investigating links between thermal tolerance and oxygen supply capacity in shark neonates from a hyperoxic tropical environment. Science of the Total Environment, 782, 146854.

Burggren, W. W., Arriaga-Bernal, J. C., Méndez-Arzate, P. M., & Méndez-Sánchez, J. F. (2019). Metabolic physiology of the Mayan cichlid fish (Mayaheros uropthalmus): Re-examination of classification as an oxyconformer. Comparative Biochemistry and Physiology Part A: Molecular & Integrative Physiology, 237, 110538.

Burggren, W. W., Mendez-Sanchez, J. F., Martinez Bautista, G., Peña, E., Martinez Garcia, R., & Alvarez Gonzalez, C. A. (2019). Developmental changes in oxygen consumption and hypoxia tolerance in the heat and hypoxia-adapted tabasco line of the Nile tilapia Oreochromis niloticus, with a survey of the metabolic literature for the genus Oreochromis. Journal of Fish Biology, 94(5), 732–744.

Butler, P. J., & Taylor, E. W. (1975). The effect of progressive hypoxia on respiration in the dogfish (Scyliorhinus canicula) at different seasonal temperatures. Journal of Experimental Biology, 63(1), 117–130.

Campos, D. F. de, Jesus, T. F., Kochhann, D., Heinrichs-Caldas, W., Coelho, M. M., & Almeida-Val, V. M. F. (2017). Metabolic rate and thermal tolerance in two congeneric Amazon fishes: Paracheirodon axelrodi Schultz, 1956 and Paracheirodon simulans Géry, 1963 (Characidae). Hydrobiologia, 789(1), 133–142.

Candebat, C. L., Booth, M., Williamson, J. E., & Pirozzi, I. (2020). The critical oxygen threshold of Yellowtail Kingfish (Seriola lalandi). Aquaculture, 516, 734519.

Capossela, K. M., Brill, R. W., Fabrizio, M. C., & Bushnell, P. G. (2012). Metabolic and cardiorespiratory responses of summer flounder Paralichthys dentatus to hypoxia at two temperatures. Journal of Fish Biology, 81(3), 1043–1058.

Chen, B.-J., Fu, S.-J., Cao, Z.-D., & Wang, Y.-X. (2019). Effect of temperature on critical oxygen tension (Pcrit) and gill morphology in six cyprinids in the Yangtze River, China. Aquaculture, 508, 137–146.

Clarke, S. B., Chapman, L. J., & Krahe, R. (2020). The effect of normoxia exposure on hypoxia tolerance and sensory sampling in a swamp-dwelling mormyrid fish. Comparative Biochemistry and Physiology Part A: Molecular & Integrative Physiology, 240, 110586.

Cochran, R. E., & Burnett, L. E. (1996). Respiratory responses of the salt marsh animals, Fundulus heteroclitus, Leiostomus xanthurus, and Palaemonetes pugio to environmental hypoxia and hypercapnia and to the organophosphate pesticide, azinphosmethyl. Journal of Experimental Marine Biology and Ecology, 195(1), 125–144.

Collins, G. M., Clark, T. D., & Carton, A. G. (2015). Physiological plasticity v. inter-population variability: Understanding drivers of hypoxia tolerance in a tropical estuarine fish. Marine and Freshwater Research, 67(10), 1575–1582.

Collins, G. M., Clark, T. D., Rummer, J. L., & Carton, A. G. (2013). Hypoxia tolerance is conserved across genetically distinct sub-populations of an iconic, tropical Australian teleost (Lates calcarifer). Conservation Physiology, 1(1).

Cook, D. G., Brown, E. J., Lefevre, S., Domenici, P., & Steffensen, J. F. (2014). The response of striped surfperch Embiotoca lateralis to progressive hypoxia: Swimming activity, shoal structure, and estimated metabolic expenditure. Journal of Experimental Marine Biology and Ecology, 460, 162–169.

Cook, D. G., Iftikar, F. I., Baker, D. W., Hickey, A. J., & Herbert, N. A. (2013). Low-O2 acclimation shifts the hypoxia avoidance behaviour of snapper (Pagrus auratus) with only subtle changes in aerobic and anaerobic function. Journal of Experimental Biology, 216(3), 369–378.

Cook, D. G., Wells, R. M., & Herbert, N. A. (2011). Anaemia adjusts the aerobic physiology of snapper (Pagrus auratus) and modulates hypoxia avoidance behaviour during oxygen choice presentations. Journal of Experimental Biology, 214(17), 2927–2934.

Corkum, C. P., & Gamperl, A. K. (2009). Does the ability to metabolically downregulate alter the hypoxia tolerance of fishes?: A comparative study using cunner (T. adspersus) and Greenland cod (G. ogac). Journal of Experimental Zoology Part A: Ecological Genetics and Physiology, 311(4), 231–239.

Craig, P. M., Fitzpatrick, J. L., Walsh, P. J., Wood, C. M., & McClelland, G. B. (2014). Coping with aquatic hypoxia: How the plainfin midshipman (Porichthys notatus) tolerates the intertidal zone. Environmental Biology of Fishes, 97(2), 163–172.

Crocker, C. E., & Cech, J. J. (1997). Effects of environmental hypoxia on oxygen consumption rate and swimming activity in juvenile white sturgeon, Acipenser transmontanus, in relation to temperature and life intervals. Environmental Biology of Fishes, 50(4), 383–389.

Cruz-Neto, A. P., & Steffensen, J. F. (1997). The effects of acute hypoxia and hypercapnia on oxygen consumption of the freshwater European eel. Journal of Fish Biology, 50(4), 759–769.

Cumming, H., & Herbert, N. A. (2016). Gill structural change in response to turbidity has no effect on the oxygen uptake of a juvenile sparid fish. Conservation Physiology, 4(1), cow033.

Dan, X.-M., Yan, G.-J., Zhang, A.-J., Cao, Z.-D., & Fu, S.-J. (2014). Effects of stable and diel-cycling hypoxia on hypoxia tolerance, postprandial metabolic response, and growth performance in juvenile qingbo (Spinibarbus sinensis). Aquaculture, 428, 21–28.

De Boeck, G., Vlaeminck, A., Van der Linden, A., & Blust, R. (2000). Salt stress and resistance to hypoxic challenges in the common carp (Cyprinus carpio L.). Journal of Fish Biology, 57(3), 761–776.

De Boeck, G., Wood, C. M., Iftikar, F. I., Matey, V., Scott, G. R., Sloman, K. A., de Nazaré Paula da Silva, M., Almeida-Val, V. M., & Val, A. L. (2013). Interactions between hypoxia tolerance and food deprivation in Amazonian oscars, Astronotus ocellatus. Journal of Experimental Biology, 216(24), 4590–4600.

Dupont-Prinet, A., Vagner, M., Chabot, D., & Audet, C. (2013). Impact of hypoxia on the metabolism of Greenland halibut (Reinhardtius hippoglossoides). Canadian Journal of Fisheries and Aquatic Sciences, 70(3), 461–469.

Ern, R., Norin, T., Gamperl, A. K., & Esbaugh, A. J. (2016). Oxygen dependence of upper thermal limits in fishes. Journal of Experimental Biology, 219(21), 3376–3383.

Fernandes, M. N., & Rantin, F. T. (1989). Respiratory responses of Oreochromis niloticus (Pisces, Cichlidae) to environmental hypoxia under different thermal conditions. Journal of Fish Biology, 35(4), 509–519.

Fitzgerald, J. A., Urbina, M. G., Rogers, N. J., Bury, N. R., Katsiadaki, I., Wilson, R. W., & Santos, E. M. (2019). Sublethal exposure to copper supresses the ability to acclimate to hypoxia in a model fish species. Aquatic Toxicology, 217, 105325.

Fitzgibbon, Q. P., Seymour, R. S., Buchanan, J., Musgrove, R., & Carragher, J. (2010). Effects of hypoxia on oxygen consumption, swimming velocity and gut evacuation in southern bluefin tuna (Thunnus maccoyii). Environmental Biology of Fishes, 89(1), 59–69.

Fu, S.-J., Brauner, C. J., Cao, Z.-D., Richards, J. G., Peng, J.-L., Dhillon, R., & Wang, Y.-X. (2011). The effect of acclimation to hypoxia and sustained exercise on subsequent hypoxia tolerance and swimming performance in goldfish (Carassius auratus). Journal of Experimental Biology, 214(12), 2080–2088.

Fu, S.-J., Fu, C., Yan, G.-J., Cao, Z.-D., Zhang, A.-J., & Pang, X. (2014). Interspecific variation in hypoxia tolerance, swimming performance and plasticity in cyprinids that prefer different habitats. Journal of Experimental Biology, 217(4), 590–597.

Geist, S. J., Ekau, W., & Kunzmann, A. (2013). Energy demand of larval and juvenile Cape horse mackerels, Trachurus capensis, and indications of hypoxia tolerance as benefit in a changing environment. Marine Biology, 160(12), 3221–3232.

Giacomin, M., Bryant, H. J., Val, A. L., Schulte, P. M., & Wood, C. M. (2019). The osmorespiratory compromise: Physiological responses and tolerance to hypoxia are affected by salinity acclimation in the euryhaline Atlantic killifish (Fundulus heteroclitus). Journal of Experimental Biology, 222(19), jeb206599.

Giacomin, M., Vilarinho, G. C., Castro, K. F., Ferreira, M., Duarte, R. M., Wood, C. M., & Val, A. L. (2018). Physiological impacts and bioaccumulation of dietary Cu and Cd in a model teleost: The Amazonian tambaqui (Colossoma macropomum). Aquatic Toxicology, 199, 30–45.

Gilmore, K. L., Doubleday, Z. A., & Gillanders, B. M. (2019). Prolonged exposure to low oxygen improves hypoxia tolerance in a freshwater fish. Conservation Physiology, 7(1), coz058.

Gordon, M. S., Belman, B. W., & Chow, P. H. (1976). Comparative studies on the metabolism of shallow-water and deep-sea marine fishes. IV. Patterns of aerobic metabolism in the mesopelagic deep-sea fangtooth fish Anoplogaster cornuta. Marine Biology, 35(3), 287–293.

Hancock, J. R., & Place, S. P. (2016). Impact of ocean acidification on the hypoxia tolerance of the woolly sculpin, Clinocottus analis. Conservation Physiology, 4(1), cow040.

Haney, D. C., & Nordlie, F. G. (1997). Influence of environmental salinity on routine metabolic rate and critical oxygen tension of Cyprinodon variegatus. Physiological Zoology, 70(5), 511–518.

Hartline, N. R., DeVries, D. R., Wright, R. A., Stoeckel, J. A., & Horne, L. M. (2020). Efiect of Temperature on Respiratory Responses to Increasing Hypoxia for Five Species of Nongame Stream Fishes. Journal of the Southeastern Association of Fish and Wildlife Agencies, 7, 93–102.

Henriksson, P., Mandic, M., & Richards, J. G. (2008). The osmorespiratory compromise in sculpins: Impaired gas exchange is associated with freshwater tolerance. Physiological and Biochemical Zoology, 81(3), 310–319.

Hermaniuk, A., van de Pol, I. L., & Verberk, W.C.E.P. (2021). Are acute and acclimated thermal effects on metabolic rate modulated by cell size? A comparison between diploid and triploid zebrafish larvae. Journal of Experimental Biology, 224(1).

Hill, J. V., Davison, W., & Marsden, I. D. (1996). Aspects of the respiratory biology of two New Zealand intertidal fishes, Acanthoclinus fuscus and Forsterygion sp. Environmental Biology of Fishes, 45(1), 85–93.

Hilton, Z., Wellenreuther, M., & Clements, K. D. (2008). Physiology underpins habitat partitioning in a sympatric sister-species pair of intertidal fishes. Functional Ecology, 22(6), 1108–1117.

Hughes, M. C., & Perry, S. F. (2021). Does blood flow limit acute hypoxia performance in larval zebrafish (Danio rerio)? Journal of Comparative Physiology B, 191(3), 469–478.

Innes, A. J., & Wells, R. M. (1985). Respiration and oxygen transport functions of the blood from an intertidal fish, Helcogramma medium (Tripterygiidae). Environmental Biology of Fishes, 14(2), 213–226.

Iversen, N. K., McKenzie, D. J., Malte, H., & Wang, T. (2010). Reflex bradycardia does not influence oxygen consumption during hypoxia in the European eel (Anguilla anguilla). Journal of Comparative Physiology B, 180(4), 495–502.

Johannsson, O. E., Giacomin, M., Sadauskas-Henrique, H., Campos, D. F., Braz-Mota, S., Heinrichs-Caldas, W. D., Baptista, R., Wood, C. M., Almeida-Val, V. M. F., & Val, A. L. (2018). Does hypoxia or different rates of re-oxygenation after hypoxia induce an oxidative stress response in Cyphocharax abramoides (Kner 1858), a Characid fish of the Rio Negro? Comparative Biochemistry and Physiology Part A: Molecular & Integrative Physiology, 224, 53–67.

Kempf, S. (2020). The physiological response of an Arctic key species Polar cod, Boreogadus saida, to environmental hypoxia: Critical oxygen level and swimming performance [PhD Thesis]. Uiniversität Bremen.

Kulesza, A., Leonard, E. M., & McClelland, G. B. (2020). Influence of 96h sub-lethal copper exposure on aerobic scope and recovery from exhaustive exercise in killifish (Fundulus heteroclitus). Aquatic Toxicology, 218, 105373.

Lapointe, D., Vogelbein, W. K., Fabrizio, M. C., Gauthier, D. T., & Brill, R. W. (2014). Temperature, hypoxia, and mycobacteriosis: Effects on adult striped bass Morone saxatilis metabolic performance. Diseases of Aquatic Organisms, 108(2), 113–127.

LeMoine, C. M., Bucking, C., Craig, P. M., & Walsh, P. J. (2014). Divergent hypoxia tolerance in adult males and females of the plainfin midshipman (Porichthys notatus). Physiological and Biochemical Zoology, 87(2), 325–333.

Malekpouri, P., Peyghan, R., Mahboobi-Soofiani, N., & Mohammadian, B. (2016). Metabolic capacities of common carp (Cyprinus carpio) following combined exposures to copper and environmental hypoxia. Ecotoxicology and Environmental Safety, 127, 1–11.

Mamun, S. M., Focken, U., & Becker, K. (2013). A respirometer system to measure critical and recovery oxygen tensions of fish under simulated diurnal fluctuations in dissolved oxygen. Aquaculture International, 21(1), 31–44.

Mandic, M., Best, C., & Perry, S. F. (2020). Loss of hypoxia-inducible factor 1α affects hypoxia tolerance in larval and adult zebrafish (Danio rerio). Proceedings of the Royal Society B, 287(1927), 20200798.

Mandic, M., Pan, Y. K., Gilmour, K. M., & Perry, S. F. (2020). Relationships between the peak hypoxic ventilatory response and critical O2 tension in larval and adult zebrafish (Danio rerio). Journal of Experimental Biology, 223(7), jeb213942.

Mandic, M., Todgham, A. E., & Richards, J. G. (2009). Mechanisms and evolution of hypoxia tolerance in fish. Proceedings of the Royal Society of London B: Biological Sciences, 276(1657), 735–744.

Mattiasen, E. G., Kashef, N. S., Stafford, D. M., Logan, C. A., Sogard, S. M., Bjorkstedt, E. P., & Hamilton, S. L. (2020). Effects of hypoxia on the behavior and physiology of kelp forest fishes. Global Change Biology, 26(6), 3498–3511.

Maxime, V., Pichavant, K., Boeuf, G., & Nonnotte, G. (2000). Effects of hypoxia on respiratory physiology of turbot, Scophthalmus maximus. Fish Physiology and Biochemistry, 22(1), 51–59.

McArley, T. J., Hickey, A. J., & Herbert, N. A. (2020). Acute high temperature exposure impairs hypoxia tolerance in an intertidal fish. PloS One, 15(4), e0231091.

McArley, T. J., Hickey, A. J., Wallace, L., Kunzmann, A., & Herbert, N. A. (2019). Intertidal triplefin fishes have a lower critical oxygen tension (Pcrit), higher maximal aerobic capacity, and higher tissue glycogen stores than their subtidal counterparts. Journal of Comparative Physiology B, 189(3), 399–411.

McBryan, T. L., Healy, T. M., Haakons, K. L., & Schulte, P. M. (2016). Warm acclimation improves hypoxia tolerance in Fundulus heteroclitus. Journal of Experimental Biology, 219(4), 474–484.

McKenzie, D. J., Lund, I., & Pedersen, P. B. (2008). Essential fatty acids influence metabolic rate and tolerance of hypoxia in Dover sole (Solea solea) larvae and juveniles. Marine Biology, 154(6), 1041–1051.

McKenzie, D. J., Steffensen, J. F., Korsmeyer, K., Whiteley, N. M., Bronzi, P., & Taylor, E. W. (2007). Swimming alters responses to hypoxia in the Adriatic sturgeon Acipenser naccarii. Journal of Fish Biology, 70(2), 651–658.

Mendez-Sanchez, J. F., & Burggren, W. W. (2017). Cardiorespiratory physiological phenotypic plasticity in developing air-breathing anabantid fishes (Betta splendens and Trichopodus trichopterus). Physiological Reports, 5(15), e13359.

Monteiro, D. A., Thomaz, J. M., Rantin, F. T., & Kalinin, A. L. (2013). Cardiorespiratory responses to graded hypoxia in the neotropical fish matrinxã (Brycon amazonicus) and traíra (Hoplias malabaricus) after waterborne or trophic exposure to inorganic mercury. Aquatic Toxicology, 140–141, 346–355. https://doi.org/10.1016/j.aquatox.2013.06.011

Moulton, T. L., Chapman, L. J., & Krahe, R. (2020). Effects of hypoxia on aerobic metabolism and active electrosensory acquisition in the African weakly electric fish Marcusenius victoriae. Journal of Fish Biology, 96(2), 496–505.

Nati, J. J., Lindström, J., Yeomans, W., & Killen, S. S. (2018). Physiological and behavioural responses to hypoxia in an invasive freshwater fish species and a native competitor. Ecology of Freshwater Fish, 27(3), 813–821.

Negrete Jr, B., & Esbaugh, A. J. (2019). A methodological evaluation of the determination of critical oxygen threshold in an estuarine teleost. Biology Open, 8(11), bio045310.

Nilsson, G. E., Hobbs, J.-P. A., & Östlund-Nilsson, S. (2007). Tribute to PL Lutz: Respiratory ecophysiology of coral-reef teleosts. Journal of Experimental Biology, 210(10), 1673–1686.

Nilsson, G. E., Hobbs, J.-P., Munday, P. L., & Östlund-Nilsson, S. (2004). Coward or braveheart: Extreme habitat fidelity through hypoxia tolerance in a coral-dwelling goby. Journal of Experimental Biology, 207(1), 33–39.

Nilsson, G. E., & Östlund-Nilsson, S. (2008). Does size matter for hypoxia tolerance in fish? Biological Reviews, 83(2), 173–189.

Nilsson, G. E., Östlund-Nilsson, S., & Munday, P. L. (2010). Effects of elevated temperature on coral reef fishes: Loss of hypoxia tolerance and inability to acclimate. Comparative Biochemistry and Physiology Part A: Molecular & Integrative Physiology, 156(4), 389–393.

Nilsson, G. E., Östlund-Nilsson, S., Penfold, R., & Grutter, A. S. (2007). From record performance to hypoxia tolerance: Respiratory transition in damselfish larvae settling on a coral reef. Proceedings of the Royal Society B: Biological Sciences, 274(1606), 79–85.

Nonnotte, G., Maxime, V., Truchot, J. P., Williot, P., & Peyraud, C. (1993). Respiratory responses to progressive ambient hypoxia in the sturgeon, Acipenser baeri. Respiration Physiology, 91(1), 71–82.

Östlund–Nilsson, S., & Nilsson, G. E. (2004). Breathing with a mouth full of eggs: Respiratory consequences of mouthbrooding in cardinalfish. Proceedings of the Royal Society of London. Series B: Biological Sciences, 271(1543), 1015–1022.

Ott, M. E., Heisler, N., & Ultsch, G. R. (1980). A re-evaluation of the relationship between temperature and the critical oxygen tension in freshwater fishes. Comparative Biochemistry and Physiology Part A: Physiology, 67(3), 337–340.

Pan, Y. K., Ern, R., Morrison, P. R., Brauner, C. J., & Esbaugh, A. J. (2017). Acclimation to prolonged hypoxia alters hemoglobin isoform expression and increases hemoglobin oxygen affinity and aerobic performance in a marine fish. Scientific Reports, 7(1), 1–11.

Pan, Y. K., Khursigara, A. J., Johansen, J. L., & Esbaugh, A. J. (2018). The effects of oil induced respiratory impairment on two indices of hypoxia tolerance in Atlantic croaker (Micropogonias undulatus). Chemosphere, 200, 143–150.

Pan, Y. K., Mandic, M., Zimmer, A. M., & Perry, S. F. (2019). Evaluating the physiological significance of hypoxic hyperventilation in larval zebrafish (Danio rerio). Journal of Experimental Biology, 222(13), jeb204800.

Paz, M. V. G., Sánchez, J. F. M., Burggren, W., & Martínez, J. L. A. G. (2020). Metabolic rate and hypoxia tolerance in Girardinichthys multiradiatus (Pisces: Goodeidae), an endemic fish at high altitude in tropical Mexico. Comparative Biochemistry and Physiology Part A: Molecular & Integrative Physiology, 239, 110576.

Pelster, B., Bridges, C. R., & Grieshaber, M. K. (1988). Respiratory adaptations of the burrowing marine teleost Lumpenus lampretaeformis (Walbaum). II. Metabolic adaptations. Journal of Experimental Marine Biology and Ecology, 124(1), 43–55.

Perna, S. A., & Fernandes, M. N. (1996). Gill morphometry of the facultative air-breathing loricariid fish, Hypostomus plecostomus (Walbaum) with special emphasis on aquatic respiration. Fish Physiology and Biochemistry, 15(3), 213–220.

Porteus, C. S., Wright, P. A., & Milsom, W. K. (2014). The effect of sustained hypoxia on the cardio-respiratory response of bowfin Amia calva: Implications for changes in the oxygen transport system. Journal of Fish Biology, 84(3), 827–843.

Rantin, F. T., Glass, M. L., Kalinin, A. L., Verzola, R. M., & Fernandes, M. N. (1993). Cardio-respiratory responses in two ecologically distinct erythrinids (Hoplias malabaricus and Hoplias lacerdae) exposed to graded environmental hypoxia. Environmental Biology of Fishes, 36(1), 93–97.

Reardon, E. E., & Chapman, L. J. (2010). Energetics of hypoxia in a mouth-brooding cichlid: Evidence for interdemic and developmental effects. Physiological and Biochemical Zoology, 83(3), 414–423.

Reardon, E. E., Parisi, A., Krahe, R., & Chapman, L. J. (2011). Energetic constraints on electric signalling in wave-type weakly electric fishes. Journal of Experimental Biology, 214(24), 4141–4150.

Reemeyer, J. E., & Rees, B. B. (2019). Standardizing the determination and interpretation of Pcrit in fishes. Journal of Experimental Biology, 222(18), jeb210633. https://doi.org/10.1242/jeb.210633

Reemeyer, J. E., & Rees, B. B. (2020). Plasticity, repeatability and phenotypic correlations of aerobic metabolic traits in a small estuarine fish. Journal of Experimental Biology, 223(14), jeb228098. https://doi.org/10.1242/jeb.228098

Regan, M. D., Gill, I. S., & Richards, J. G. (2017a). Calorespirometry reveals that goldfish prioritize aerobic metabolism over metabolic rate depression in all but near-anoxic environments. Journal of Experimental Biology, 220(4), 564–572.

Regan, M. D., Gill, I. S., & Richards, J. G. (2017b). Metabolic depression and the evolution of hypoxia tolerance in threespine stickleback, Gasterosteus aculeatus. Biology Letters, 13(11), 20170392.

Regan, M. D., Kuchel, L. J., Huang, S. S., Higgs, D. A., Wang, J., Schulte, P. M., & Brauner, C. J. (2010). The effect of dietary fish oil and poultry fat replacement with canola oil on swimming performance and metabolic response to hypoxia in stream type spring Chinook salmon parr. Aquaculture, 308(3–4), 183–189.

Regan, M. D., & Richards, J. G. (2017). Rates of hypoxia induction alter mechanisms of O2 uptake and the critical O2 tension of goldfish. Journal of Experimental Biology, 220(14), 2536–2544.

Remen, M., Oppedal, F., Imsland, A. K., Olsen, R. E., & Torgersen, T. (2013). Hypoxia tolerance thresholds for post-smolt Atlantic salmon: Dependency of temperature and hypoxia acclimation. Aquaculture, 416, 41–47.

Richards, J. G., Sardella, B. A., & Schulte, P. M. (2008). Regulation of pyruvate dehydrogenase in the common killifish, Fundulus heteroclitus, during hypoxia exposure. American Journal of Physiology-Regulatory, Integrative and Comparative Physiology, 295(3), R979–R990.

Rodgers, E. M., Opinion, A. G. R., Isaza, D. F. G., Rašković, B., Poleksić, V., & De Boeck, G. (2021). Double whammy: Nitrate pollution heightens susceptibility to both hypoxia and heat in a freshwater salmonid. Science of The Total Environment, 765, 142777.

Rosenberger, A. E., & Chapman, L. J. (2000). Respiratory characters of three species of haplochromine cichlids: Implications for use of wetland refugia. Journal of Fish Biology, 57(2), 483–501.

Routley, M. H., Nilsson, G. E., & Renshaw, G. M. (2002). Exposure to hypoxia primes the respiratory and metabolic responses of the epaulette shark to progressive hypoxia. Comparative Biochemistry and Physiology Part A: Molecular & Integrative Physiology, 131(2), 313–321.

Saint-Paul, U. (1984). Physiological adaptation to hypoxia of a neotropical characoid fish Colossoma macropomum, Serrasalmidae. Environmental Biology of Fishes, 11(1), 53–62.

Sakuragui, M. M., Sanches, J. R., & Fernandes, M. N. (2003). Gill chloride cell proliferation and respiratory responses to hypoxia of the neotropical erythrinid fish Hoplias malabaricus. Journal of Comparative Physiology B, 173(4), 309–317.

Schjolden, J., Sørensen, J., Nilsson, G. E., & Poléo, A. B. (2007). The toxicity of copper to crucian carp (Carassius carassius) in soft water. Science of the Total Environment, 384(1–3), 239–251.

Schofield, P. J., & Chapman, L. J. (2000). Hypoxia tolerance of introduced Nile perch: Implications for survival of indigenous fishes in the Lake Victoria basin. African Zoology, 35(1), 35–42.

Schurmann, H., & Steffensen, J. F. (1997). Effects of temperature, hypoxia and activity on the metabolism of juvenile Atlantic cod. Journal of Fish Biology, 50(6), 1166–1180.

Schwieterman, G. D., Crear, D. P., Anderson, B. N., Lavoie, D. R., Sulikowski, J. A., Bushnell, P. G., & Brill, R. W. (2019). Combined Effects of Acute Temperature Change and Elevated pCO2 on the Metabolic Rates and Hypoxia Tolerances of Clearnose Skate (Rostaraja eglanteria), Summer Flounder (Paralichthys dentatus), and Thorny Skate (Amblyraja radiata). Biology, 8(3), 56.

Shi, K., Dong, S., Zhou, Y., Gao, Q., Li, L., Zhang, M., & Sun, D. (2018). Comparative evaluation of toleration to heating and hypoxia of three kinds of salmonids. Journal of Ocean University of China, 17(6), 1465–1472.

Sloman, K. A., Sloman, R. D., De Boeck, G., Scott, G. R., Iftikar, F. I., Wood, C. M., Almeida-Val, V. M., & Val, A. L. (2009). The role of size in synchronous air breathing of Hoplosternum littorale. Physiological and Biochemical Zoology, 82(6), 625–634.

Sloman, K. A., Wood, C. M., Scott, G. R., Wood, S., Kajimura, M., Johannsson, O. E., Almeida-Val, V. M., & Val, A. L. (2006). Tribute to RG Boutilier: The effect of size on the physiological and behavioural responses of oscar, Astronotus ocellatus, to hypoxia. Journal of Experimental Biology, 209(7), 1197–1205.

Smit, H., Van Den Berg, R. J., Kijne-Den Hartog, I., & Rozing, J. (1973). Some experiments on thermal acclimation in the goldfish (Carassius auratus L.). Netherlands Journal of Zoology, 24(1), 32–49.

Snyder, S., Nadler, L. E., Bayley, J. S., Svendsen, M. B. S., Johansen, J. L., Domenici, P., & Steffensen, J. F. (2016). Effect of closed v. Intermittent-flow respirometry on hypoxia tolerance in the shiner perch Cymatogaster aggregata. Journal of Fish Biology, 88(1), 252–264.

Sollid, J., Weber, R. E., & Nilsson, G. E. (2005). Temperature alters the respiratory surface area of crucian carp Carassius carassius and goldfish Carassius auratus. Journal of Experimental Biology, 208(6), 1109–1116.

Sørensen, C., Munday, P. L., & Nilsson, G. E. (2014). Aerobic vs. anaerobic scope: Sibling species of fish indicate that temperature dependence of hypoxia tolerance can predict future survival. Global Change Biology, 20(3), 724–729.

Speers-Roesch, B., Richards, J. G., Brauner, C. J., Farrell, A. P., Hickey, A. J., Wang, Y. S., & Renshaw, G. M. (2012). Hypoxia tolerance in elasmobranchs. I. Critical oxygen tension as a measure of blood oxygen transport during hypoxia exposure. The Journal of Experimental Biology, 215(1), 93–102.

Steffensen, J. F., Bushnell, P. G., & Schurmann, H. (1994). Oxygen consumption in four species of teleosts from Greenland: No evidence of metabolic cold adaptation. Polar Biology, 14(1), 49–54.

Steffensen, J. F., Lomholt, J. P., & Johansen, K. (1982). Gill ventilation and O 2 extraction during graded hypoxia in two ecologically distinct species of flatfish, the flounder (Platichthys flesus) and the plaice (Pleuronectes platessa). Environmental Biology of Fishes, 7(2), 157–163.

Stevens, E. D., Sutterlin, A., & Cook, T. (1998). Respiratory metabolism and swimming performance in growth hormone transgenic Atlantic salmon. Canadian Journal of Fisheries and Aquatic Sciences, 55(9), 2028–2035.

Stoffels, R. J. (2015). Physiological trade-offs along a fast-slow lifestyle continuum in fishes: What do they tell us about resistance and resilience to hypoxia? PLoS One, 10(6), e0130303.

Stoffels, R. J., Weatherman, K. E., & Allen-Ankins, S. (2017). Heat and hypoxia give a global invader, Gambusia holbrooki, the edge over a threatened endemic fish on Australian floodplains. Biological Invasions, 19(8), 2477–2489.

Svendsen, J. C., Steffensen, J. F., Aarestrup, K., Frisk, M., Etzerodt, A., & Jyde, M. (2012). Excess posthypoxic oxygen consumption in rainbow trout (Oncorhynchus mykiss): Recovery in normoxia and hypoxia. Canadian Journal of Zoology, 90(1), 1–11.

Takasusuki, J., Fernandes, M. N., & Severi, W. (1998). The occurrence of aerial respiration in Rhinelepis strigosa during progressive hypoxia. Journal of Fish Biology, 52(2), 369–379.

Thomaz, J. M., Martins, N. D., Monteiro, D. A., Rantin, F. T., & Kalinin, A. L. (2009). Cardio-respiratory function and oxidative stress biomarkers in Nile tilapia exposed to the organophosphate insecticide trichlorfon (NEGUVON®). Ecotoxicology and Environmental Safety, 72(5), 1413–1424. https://doi.org/10.1016/j.ecoenv.2008.11.003

Thuy, N. H., Tien, L. A., Tuyet, P. N., Huong, D. T. T., Cong, N. V., Bayley, M., Wang, T., & Lefevre, S. (2010). Critical oxygen tension increases during digestion in the perch Perca fluviatilis. Journal of Fish Biology, 76(4), 1025–1031. https://doi.org/10.1111/j.1095-8649.2009.02533.x

Tiffany, B. N., Enzor, L. A., & Bennett, W. A. (2010). Responses of skilletfish Gobiesox strumosus to high temperature and low oxygen stress. Journal of Fish Biology, 76(3), 556–563. https://doi.org/10.1111/j.1095-8649.2009.02511.x

Timmerman, C. M., & Chapman, L. J. (2004a). Behavioral and physiological compensation for chronic hypoxia in the sailfin molly (Poecilia latipinna). Physiological and Biochemical Zoology, 77(4), 601–610.

Timmerman, C. M., & Chapman, L. J. (2004b). Hypoxia and interdemic variation in Poecilia latipinna. Journal of Fish Biology, 65(3), 635–650.

Tripathi, R. K., Mohindra, V., Singh, A., Kumar, R., Mishra, R. M., & Jena, J. K. (2013). Physiological responses to acute experimental hypoxia in the air-breathing Indian catfish, Clarias batrachus (Linnaeus, 1758). Journal of Biosciences, 38(2), 373–383.

Ultsch, G. R., Boschung, H., & Ross, M. J. (1978). Metabolism, critical oxygen tension, and habitat selection in darters (Etheostoma). Ecology, 59(1), 99–107.

Ultsch, G. R., Jackson, D. C., & Moalli, R. (1981). Metabolic oxygen conformity among lower vertebrates: The toadfish revisited. Journal of Comparative Physiology, 142(4), 439–443. https://doi.org/10.1007/BF00688973

Urbina, M. A., Glover, C. N., & Forster, M. E. (2012). A novel oxyconforming response in the freshwater fish Galaxias maculatus. Comparative Biochemistry and Physiology Part A: Molecular & Integrative Physiology, 161(3), 301–306.

Valverde, J. C., López, F.-J. M., & García, B. G. (2006). Oxygen consumption and ventilatory frequency responses to gradual hypoxia in common dentex (Dentex dentex): Basis for suitable oxygen level estimations. Aquaculture, 256(1–4), 542–551.

Wells, R. M. G. (1987). Respiration of Antarctic fish from McMurdo Sound. Comparative Biochemistry and Physiology Part A: Physiology, 88(3), 417–424. https://doi.org/10.1016/0300-9629(87)90056-9

Wong, C. C., Drazen, J. C., Callan, C. K., & Korsmeyer, K. E. (2018). Hypoxia tolerance in coral-reef triggerfishes (Balistidae). Coral Reefs, 37(1), 215–225.

Yamanaka, H. (2007). Difference in the hypoxia tolerance of the round crucian carp and largemouth bass: Implications for physiological refugia in the macrophyte zone. Ichthyological Research, 54(3), 308–312. https://doi.org/10.1007/s10228-006-0400-0

Yamanaka, H., Takahara, T., Kohmatsu, Y., & Yuma, M. (2013). Body size and temperature dependence of routine metabolic rate and critical oxygen concentration in larvae and juveniles of the round crucian carp Carassius auratus grandoculis Temminck & Schlegel 1846. Journal of Applied Ichthyology, 29(4), 891–895. https://doi.org/10.1111/jai.12126

Yang, Y., Cao, Z.-D., & Fu, S.-J. (2015). Variations in temperature acclimation effects on glycogen storage, hypoxia tolerance and swimming performance with seasonal acclimatization in juvenile Chinese crucian carp. Comparative Biochemistry and Physiology Part A: Molecular & Integrative Physiology, 185, 16–23.

Zhang, Y., Healy, T. M., Vandersteen, W., Schulte, P. M., & Farrell, A. P. (2018). A rainbow trout Oncorhynchus mykiss strain with higher aerobic scope in normoxia also has superior tolerance of hypoxia. Journal of Fish Biology, 92(2), 487–503.
