# Supplementary material for: Body mass and cell size shape the tolerance of fishes to low oxygen in a temperature‐dependent manner
Source: Glob Chang Biol. 2022 Jul 25;28(19):5695–707. doi: 10.1111/gcb.16319 (PMC9542040; doi:10.1111/gcb.16319)
Supplement: Supplementary file 2 — Appendix S2 [file GCB-28-5695-s002.docx]

Supplementary material to:

Body mass and cell size shape the tolerance of fishes to low oxygen in a temperature-dependent manner

Wilco C.E.P. Verberk^1^, Jeroen F. Sandker^1^, Iris van de Pol^1^, Mauricio Urbina^2,3^, Rod W. Wilson^4^, David J. McKenzie^5^ and Félix P. Leiva^1^

1. Department of Animal Ecology and Physiology, Radboud Institute for Biological and Environmental Sciences, Radboud University Nijmegen, PO Box 9010, 6500 GL Nijmegen, The Netherlands.

2. Departamento de Zoología, Facultad de Ciencias Naturales y Oceanográficas, Universidad de Concepción, Casilla 160-C, Concepción, Chile.

3. Instituto Milenio de Oceanografía (IMO), Universidad de Concepción, Chile.

4. Biosciences, University of Exeter, Exeter, UK.

5. MARBEC, Univ Montpellier, CNRS, IFREMER, IRD, Montpellier, France.


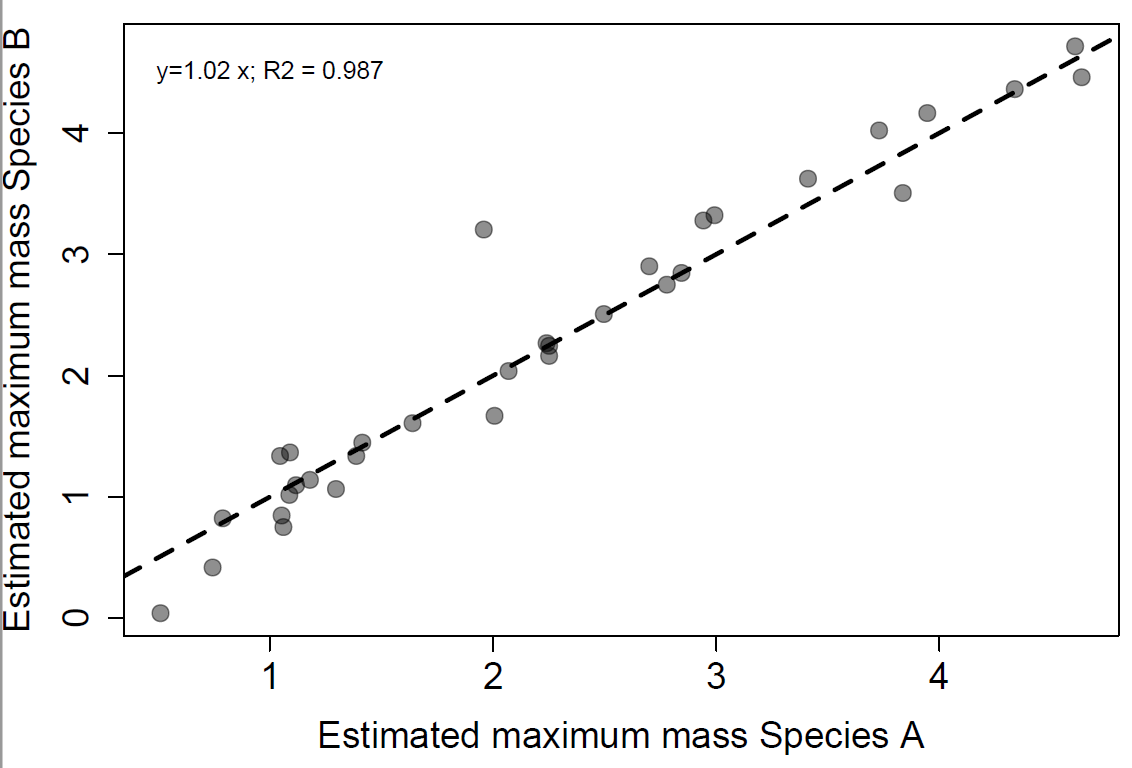


Figure S1. Estimated maximum mass (log10 transformed) for pairs of closely related species. Each point represents a pair of two closely related species (species A and B) for which the maximum mass was estimated based on its documented maximum length and the length-weight conversion for that species. The dotted line equals y=x.


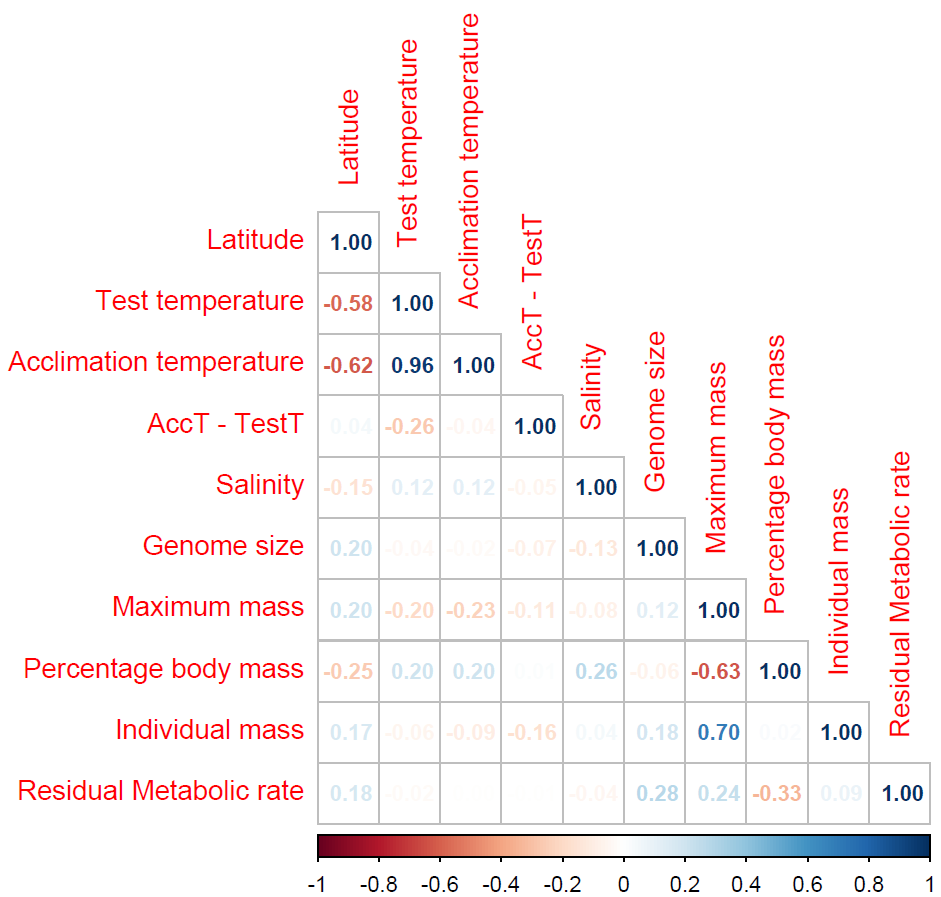


Figure S2. Correlation plot between the continuous predictor variables, showing spearman rank correlation coefficients.


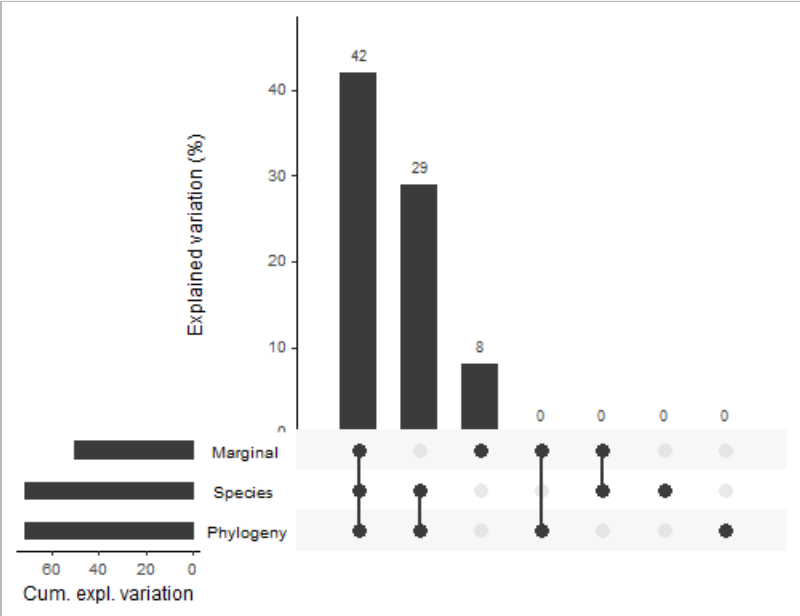
Figure S3. Variance partitioning graph showing the relative contributions of marginal effects, species effects and phylogeny effects. Variances are ranked from the highest (shared variance between all three categories) to the lowest (unique contribution of Phylogeny).


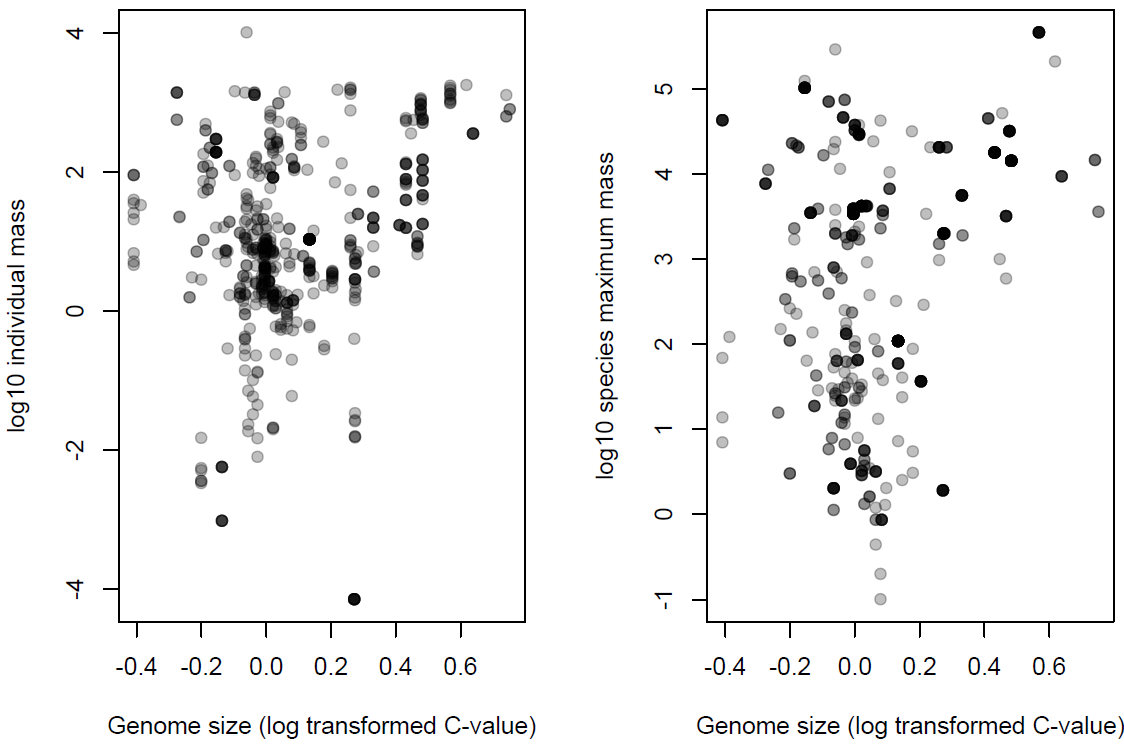
Figure S4. Plots of the raw data between genome size and individual body mass (left) and maximum body mass (right). Spearman rank correlations showed weak, but significant effects, but when analysed within a phylogenetic context, no significant relationships were found.

| Number | A1 | A2 | A3 | A4 | A5 | A6 | A7 | A8 | B1 | B2 | B3 | B4 | B5 | B6 | B7 | B8 | C1 | C2 |
| --- | --- | --- | --- | --- | --- | --- | --- | --- | --- | --- | --- | --- | --- | --- | --- | --- | --- | --- |
| Phylogeny | x | x | x | x | x | x | x | x | x | X | x | x | x | x | x | x | x | x |
| Species | x | x | x | x | x | x | x | x |  |  |  |  |  |  |  |  | x |  |
| Measurement temperature | x | x | x | x | x | x | x | x | x | x | x | x | x | x | x | x | x | x |
| Acclimation temperature | x | x | x | x | x | x | x | x | x | x | x | x | x | x | x | x |  |  |
| Salinity | x | x | x | x | x | x | x | x | x | x | x | x | x | x | x | x | x | x |
| Respirometry type | x | x | x | x | x | x | x | x | x | x | x | x | x | x | x | x |  |  |
| Residual metabolic rate | x | x | x | x | x | x | x | x | x | x | x | x | x | x | x | x | x | x |
| Genome size |  | x |  | x | x | x | x | x |  | x |  | x |  | x | x | x | x | x |
| Body mass | x |  |  | x |  | x | x | x | x |  |  | x |  | x | x | x | x | x |
| Relative body mass |  |  | x |  | x | x | x | x |  |  | x |  | x | x | x | x |  |  |
| Relative body mass^2 |  |  |  |  |  |  |  | x |  |  |  |  |  |  |  | x |  |  |
| Measurement temperature:Body mass | x |  |  | x |  | x | x | x | x |  |  | x |  | x | x | x | x | x |
| Measurement temperature:Genome size |  | x |  | x | x | x | x | x |  | x |  | x |  | x | x | x | x | x |
| Measurement temperature:Relative body mass |  |  | x |  | x |  | x |  |  |  | x |  | x | x |  |  |  |  |
| looic | 2419.4 | 2408.7 | 2422.9 | 2380.4 | 2385.2 | 2359.7 | 2382.5 | 2360.1 | 2418.4 | 2406.0 | 2422.3 | 2381.2 | 2380.4 | 2359.7 | 2382.4 | 2363.1 | 2427.6 | 2428.9 |
| p_loo | 101.3 | 105.5 | 99.8 | 108.4 | 110.1 | 110.2 | 109.5 | 109.8 | 98.7 | 102.0 | 98.2 | 105.4 | 105.1 | 105.8 | 105.8 | 107.3 | 106.3 | 102.6 |
| elpd_loo | -1209.7 | -1204.4 | -1211.5 | -1190.2 | -1192.6 | -1179.8 | -1191.2 | -1180.0 | -1209.2 | -1203.0 | -1211.2 | -1190.6 | -1190.2 | -1179.9 | -1191.2 | -1181.6 | -1213.8 | -1214.4 |
| elpd_loo_SE | 26.2 | 26.2 | 25.3 | 27.6 | 27.0 | 28.3 | 27.9 | 27.9 | 26.1 | 26.1 | 25.3 | 27.6 | 26.6 | 27.7 | 27.8 | 27.8 | 27.0 | 27.2 |
| elpd_loo_diff | -29.9 | -24.5 | -31.6 | -10.3 | -12.8 | 0.0 | -11.4 | -0.2 | -29.3 | -23.2 | -31.3 | -10.8 | -10.3 | 0.0 | -11.3 | -1.7 | 0.0 | -0.6 |
| model support | 0.0000 | 0.0000 | 0.0000 | 0.0000 | 0.0000 | 0.5483 | 0.0000 | 0.4517 | 0.0000 | 0.0000 | 0.0000 | 0.0000 | 0.0000 | 0.8475 | 0.0000 | 0.1525 | 0.6490 | 0.3510 |

Table S1. List of all models using Pareto-smoothing importance sampling leave-one-out cross validation (PSIS-LOO). Models A1-A8 include two random effects, one over species given the variance-covariance matrix of the phylogenetic tree and the other one as an unstructured random species effect, which accounted for any specific effect that would be independent of the phylogenetic relationship between species. Models B1-B8 only include the phylogenetic variance-covariance matrix as a random effect. For Models C1 and C2, the best performing models for A and B were simplified by excluding acclimation temperature (not always signifcant) and Respirometry type (not informative). For each model, the loo package was used to calculate the LOO information criterion value (looic), the effective number of parameters (ploo), the expected log predictive density (elpdloo) and its standard error, the difference in the expected log predictive density (elpddiff) when compared to the best model. Model support is calculated as the Bayesian stacking weight (similar to Akaike weight) and the model with most support is highlighted in grey.

Table S2. Models comparison using Pareto-smoothing importance sampling leave-one-out cross validation (PSIS-LOO). Model Genome size has a similar structure as model C1 in Table S1, while Model Maximum body mass effects of genome size are substituted by maximum body mass. Note that both models are based on 594 records (i.e. 6 records less than those in Table S1 since we could not retrieve maximum body mass in those 6 cases).

| Number | Genome size | Maximum body mass |
| --- | --- | --- |
| Phylogeny | x | x |
| Species | x | x |
| Measurement temperature | x | x |
| Salinity | x | x |
| Residual metabolic rate | x | x |
| Genome size | x |  |
| Body mass | x | x |
| Maximum body mass |  | x |
| Measurement temperature:Body mass | x | x |
| Measurement temperature:Genome size | x |  |
| Measurement temperature:max body mass |  | x |
| looic | 2406.4 | 2439.3 |
| p_loo | 106.6 | 94.9 |
| elpd_loo | -1203.2 | -1219.6 |
| elpd_loo_SE | 27.1 | 25.5 |
| elpd_loo_diff | 0.0 | -16.4 |
| model support | 1.0000 | 0.0000 |
